# Curve Fitting of the Corporate Recovery Rates: The Comparison of Beta Distribution Estimation and Kernel Density Estimation

**DOI:** 10.1371/journal.pone.0068238

**Published:** 2013-07-10

**Authors:** Rongda Chen, Ze Wang

**Affiliations:** 1 School of Finance, Zhejiang University of Finance and Economics, Hangzhou, China; 2 Coordinated Innovation Center of Wealth Management and Quantitative Investment of Zhejiang University of Finance and Economics, Hangzhou, China; 3 Center for Research of Regulation and Policy of Zhejiang Province, Hangzhou, China; University of Florida, United States of America

## Abstract

Recovery rate is essential to the estimation of the portfolio’s loss and economic capital. Neglecting the randomness of the distribution of recovery rate may underestimate the risk. The study introduces two kinds of models of distribution, Beta distribution estimation and kernel density distribution estimation, to simulate the distribution of recovery rates of corporate loans and bonds. As is known, models based on Beta distribution are common in daily usage, such as CreditMetrics by J.P. Morgan, Portfolio Manager by KMV and Losscalc by Moody’s. However, it has a fatal defect that it can’t fit the bimodal or multimodal distributions such as recovery rates of corporate loans and bonds as Moody’s new data show. In order to overcome this flaw, the kernel density estimation is introduced and we compare the simulation results by histogram, Beta distribution estimation and kernel density estimation to reach the conclusion that the Gaussian kernel density distribution really better imitates the distribution of the bimodal or multimodal data samples of corporate loans and bonds. Finally, a Chi-square test of the Gaussian kernel density estimation proves that it can fit the curve of recovery rates of loans and bonds. So using the kernel density distribution to precisely delineate the bimodal recovery rates of bonds is optimal in credit risk management.

## Introduction

Credit risk is the distribution of financial losses caused by unexpected changes in compliance of financial agreements. The recovery rate is an important measure of how much we can retrieve from bad debts. So it is crucial to figure out what kind of distribution recovery rates of the sample comply with. An important key in building the credit risk model is the recovery rate in default or loss given default (LGD) function, expressed as a ratio (dollar recovery/amount invested) [1].

Recently, research on recovery rate has mainly focused on factors that impact recovery rate, correlation between recovery rate and default rate, its distribution, etc. [2]. Compared with default rate, the influencing factors of recovery rate are more complex. The most representative model is Losscalc Model of Moody’s. The correlation between recovery rate and default rate turns out to be positive. Hu & Perraudin [3], Rosch & Scheule [4] find that it will underestimate the portfolio’s loss if the correlation is neglected.

Histogram is common and is widely used to depict the distribution, but it can’t be a smooth curve [5]. Beta distribution estimation is widely used for simulating the curve of the recovery rates [6]. Besides, Frye [7] assumes that the recovery rate follows a normal distribution and Pykhtin [8] establishes a log-normal distribution and Andersen & Sidennius [9] discuss the probit-normal distribution. Dullmann & Trapp [10] utilize a logit-normal distribution and empirically analyse the recovery rates. After comparing the results with two other extended models, Frye [7] and Pykhtin [8], the log-normal distribution is not found to be as good as the other two distributions.

How to precisely depict the randomness of recovery rate is essential to the estimation of the loss of portfolio and economic capital. In this paper, we mainly focus on distribution of recovery rate. Beta distribution is widely used in many credit risk models such as CreditMetrics by J.P. Morgan, Portfolio Manager by KMV and Losscalc by Moody’s. However, sometimes it ceases to be effective because of the fact that the recovery rates curve always has two peaks while Beta distribution estimation can only demonstrate one peak. So in this paper we adopt the Gaussian kernel density estimation, a nonparametric estimation, to solve this problem and justify our hypothesis. Kernel density estimation put forward by Emanuel Parzen (1955) and Murray Rosenblatt (1962),also named as the Parzen-Rosenblatt window method, is a non-parametric way to estimate the probability density function of a random variable. Ruppert and Cline proposed revision of the kernel density estimation method based upon data density function and clustering algorithm. We can build the prediction model of value at risk through the kernel density estimation of a random variable. Moreover, weighted processing of the estimated variation coefficient can help build different risk prediction models. In order to avoid producing the setting model error caused by the preset distribution of stock returns, Liu & He [11] used the kernel density estimation to fit stock returns, and then tested the result by Monte Carlo simulation. Their study shows kernel density estimation result is a good approximation of the real stock returns distribution. Zhen & Li [12] study the distribution of Hang Seng index returns by the nonparametric kernel density estimation, which exhibits the fat tail characteristic. Shi & Huang [13] use the kernel density estimation method to analyze the dynamic economic growth at the provincial level in China from 1978 to 2007. Their research indicates that the growth distribution at provincial level has the trend of bimodal distribution, which is especially obvious in 1999. In this paper, we use kernel density estimation to fit the distribution of the bimodal or multimodal samples of recovery rates of corporate loans and bonds.

This paper is organized as follows. In Part 2.1 the recovery rate and sources of data are introduced. Part 2.2 introduces the Beta distribution estimation method [6] with five different shapes. In Part 2.3 we introduce properties of estimation of the kernel density and several ways to figure out the bandwidth of the kernel density. In Part 2.4, the Chi-square test is introduced [14]. Part 3 shows the results. It contains the study of fitting the curves to the recovery rates of corporate loans and bonds (from Moody’s Investors Service) for the years 2009–2011 by histogram, Beta distribution estimation and kernel density estimation. We favor Beta distribution estimation over histogram in our study. Kernel density estimation depicts the bimodal or multimodal distributions of recovery rates while beta distribution estimation cannot. In addition, we test whether the kernel density estimation can fit the distribution of the recovery rate. Part 4 concludes.

## Materials and Methods

### 1. Data of Recovery Rate

Recovery is usually measured by two indicators: the ultimate recovery rate – value of assets that the creditors eventually retrieve, and the price of debt after default. Prices of defaulted assets only exist in the defaultable security market in public, but some of the tradable bonds can also offer the data of their prices after default in private. In addition, default debt prices should be discounted, however, as default bonds generally lack liquidity, it is very difficult to calculate the ultimate recovery rates. In this paper we collect the data of recovery rates of corporate loans from year 2010 to year 2011 [15], [16] and those of corporate bonds from year 2009 to year 2011 for analysis.

### 2. Beta Distribution Estimation

The common Beta density function is defined as follows:
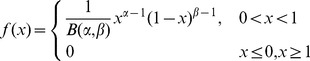
(1)Where 
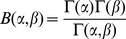
,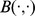 means a Beta function, 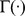 means a Gamma function.

The shape of Beta distribution is based on values of parameters 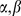.

1. *if 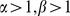, the distribution is bell shaped;*
2. *if 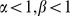, the distribution is U shaped;*
3. *if 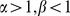 the distribution is J shaped;*
4. *if 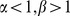, the distribution is inverse J shaped;*
5. *if 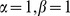, the distribution is an uniform distribution;*

**Figure 1:**
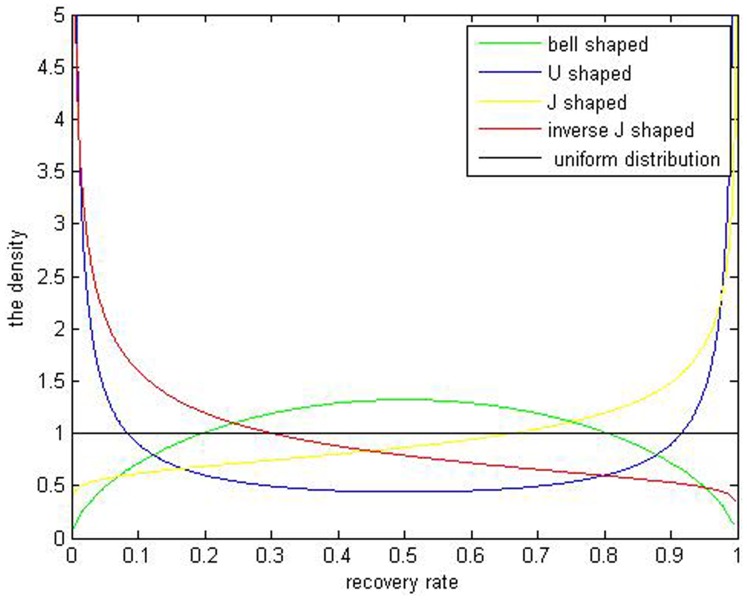
Beta distribution curves are different for the different input of the parameters 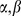. The bell shaped curve is simulated by 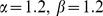. The U shaped curve is simulated by 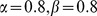. The J shaped curve is simulated by 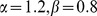. The inverse J shaped curve is simulated by 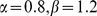. The uniform distribution is simulated by 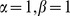.

Figure 1 Shows the different shapes of Beta distribution from simulation by Matlab7.6 software.

The relationships between statistics such as mean 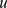 and variance 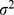 and the parameters 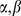 are stated as follows:
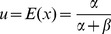
(2)

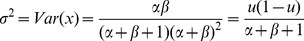
(3)


### 3. Kernel Density Estimation

#### 3.1 Definition of kernel density estimation

Kernel density estimation is a particular nonparametric technique to estimate the underlying density as a weighted average of local functions centered at each sample point. It can asymptotically converge to any density function. Suppose 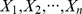 are samples from population 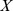, n is the number of samples. If there is a bounded function 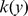 satisfying: *1)* 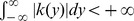 *; 2) 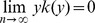;* *3)* 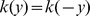 *4) 
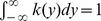
;* The
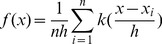
(4)

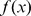
 is the kernel density estimation, where *K* is a kernel function, *h*>0 is a smoothing parameter called the bandwidth and *n* is the number of samples. There are many common types of kernel function in Table 1.

**Table 1:** Expressions of common kernel density functions.

Kernel density function	Uniform	Triangle	Gaussian	Epanchnikov	Double exponential	Cosine arch
equation	U(−1,1)	(1−|t|)	N(0,1)	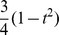	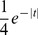	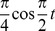

In this paper, we choose the Gaussian kernel density, and its expression is
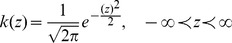
(5)


Putting Equation (5) into Equation (4), we have
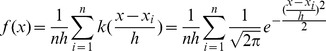
(6)


Equation (6) is the final Gaussian kernel density function.

#### 3.2 Bandwidth estimation

The bandwidth *h* plays a paramount role in the estimation compared with the form of *K*. Martin [17] believes that if *h* is large, the estimation will be over-smooth, and vice versa. There are three main standards to decide the bandwidth

*1) Mean square error, MSE*



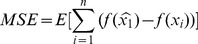
(7)
*2) Integral mean square error is adopted when the density is continuous, MISE*



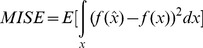
(8)
*3) Asymptotic integral mean square error, AMISE*



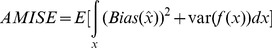
(9)Also there are many other methods to choose the bandwidth. Most of these methods are based on the idea of minimizing the MSE or the MISE. The following methods [18] are often used to select the bandwidth for:

*4) Least-squares cross-validation.*

*5) Biased cross-validation.*

*6) Plug-in bandwidth selection.*

*7) Smoothed cross-validation*

*8) Root-n bandwidth selection*

*9) The Contrast Metho*d

As *n* become larger, *h* becomes smaller. However, if *h* is too small, it reduces the accuracy of the estimation. On the other hand, if *h* is too large, its estimated curve will be too smooth. In most cases, MSE and MISE are used most commonly.

### 4. Chi-square Goodness-of-fit Test

In order to assess whether the qualitative data fit the kernel density distribution, We adopt Chi-square goodness-of-fit test to test the results. The test is performed by grouping the data into bins, calculating the observed and expected counts for the bins, and computing the chi-square test statistic shown as
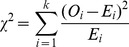
(10)Where k means the sample can be divided into k bins or intervals, 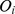 are the observed counts and 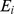 are the expected counts in bin *i*. Suppose 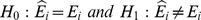. The null hypothesis means that the frequency in each interval equals that of corresponding kernel distribution. If the p-value is below the significance level (we often choose 5% or 10%), the null hypothesis can be rejected and the kernel density estimation doesn’t quite fit the curve of the recovery rates of municipal bonds. If the result is above the significance level, we have no reason to reject the null hypothesis
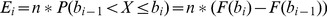
(11)


Where 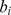 stand for the value of bin edges, n is the number of observations in the sample. 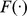 is the cumulative density function of the kernel distribution.

## Results

### 1. Histograms of Recovery Rates

By sorting the data from the Moody’s analysis report, we get 58 effective observations of recovery rates of corporate loans from year 2010 to 2011 and 282 ones of corporate bonds from year 2009 to 2011. The results are shown in Table 2.

**Table 2:** Statistics of sample recovery rates.

	Mean	Variance	Skewness	Kurtosis
Recovery rates of defaulted corporate loans from year 2010 to 2011	0.5574	0.1003	−0.1706	1.5603
Recovery rates of defaulted corporate bonds from year 2009 to 2011	0.3895	0.0898	0.4672	2.0161

**Figure 2:**
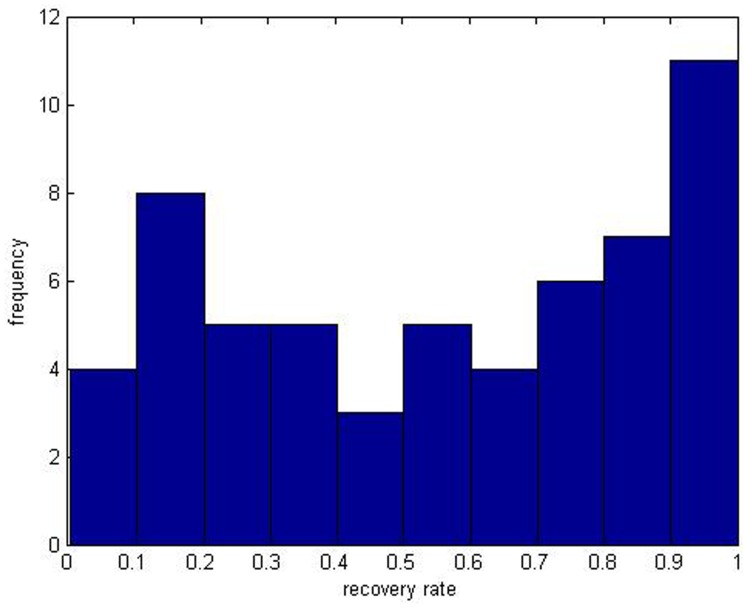
It is the histogram of 58 effective observations of recovery rates of corporate loans from year 2010 to 2011. The data comes from the Moody’s analysis report.

**Figure 3:**
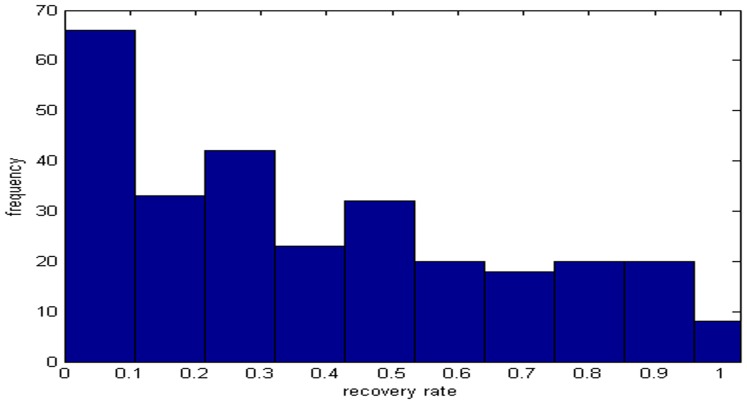
It is the histogram of 282 observations of recovery rates of corporate bonds from year 2009 to 2011. The data comes from the Moody’s analysis report.

Table 2 exhibits the main characteristics of the sample of recovery rates. Meanwhile their histograms are given as follows (See Fig. 2 and Fig. 3).

The histogram of defaulted loans’ recovery rates (Fig. 2) demonstrates two peaks, where a bimodal characteristic can be seen that the probabilities of full recovery rates ranging from 0.9 to 1 and low ones from 0.1 to 0.2 are both very high. Also Figure 3 shows that 4 peaks exist at the intervals of 0 to 0.1, 0.2 to 0.3, 0.4 to 0.5 and 0.8 to 0.9. As we know, the number of default events has increased since the financial crisis, and individuals have different abilities to repay their loans and bonds. However, it used to be rare to have so many default events.

### 2. Beta Distribution Estimation of the Recovery Rate

**Figure 4:**
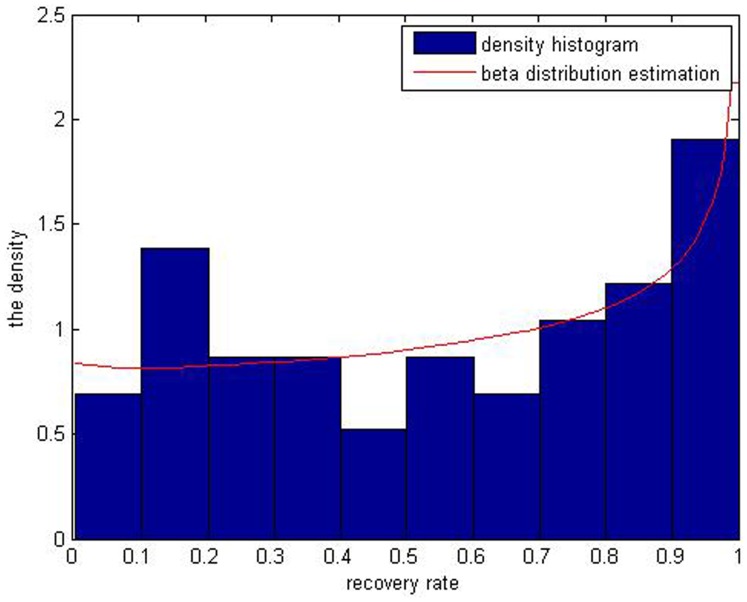
The red curve the result of Beta simulation of defaulted corporate loans’ recovery rates. It well simulates the bimodal distribution of the recovery rates.

**Figure 5:**
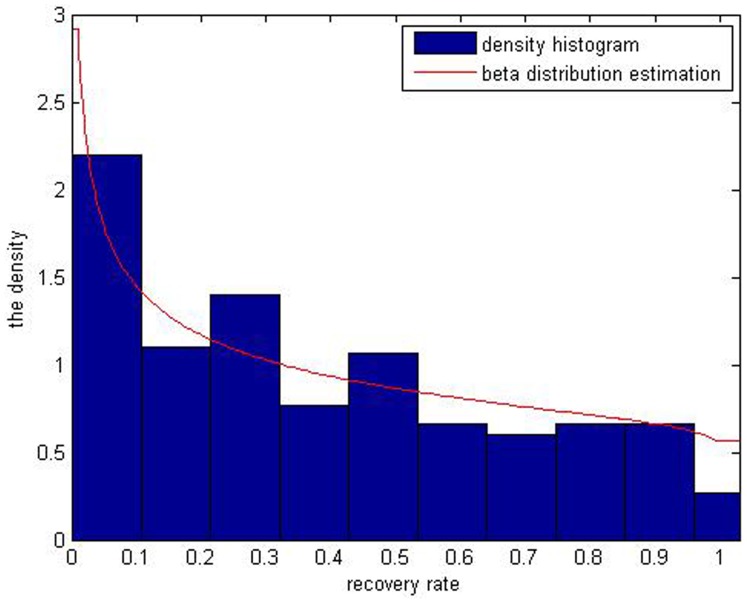
The red curve the result of Beta simulation of defaulted corporate bonds’ recovery rates. It well simulates the multimodal distribution of the recovery rates.

A common method to estimate the distribution of recovery rates is Beta distribution, which forms a smooth curve compared with the histogram. Through the data of recovery rates of defaulted corporate loans, we get the mean which equals 0.5574 and the variance is 0.1003. Inputting the outcome above into the Equations (2) and (3) leads to values of parameters 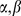, being 0.9797 and 0.7712, respectively. By Matlab7.6 software [19], we get the simulated distribution of the real recovery rate (Figure 4). The Beta distribution estimation cannot fit the bimodal distribution of defaulted loans’ recovery rates. Meanwhile, we can get the value of parameters 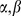 of defaulted bonds’ recovery rates, which are 0.9797 and 0.7712 respectively. Besides, we get simulated distribution of recovery rates in Figure 5. It also illustrates that Beta distribution estimation can partly describe the distribution of recovery rates but cannot fit its multiple peaks characteristic.

As is shown above, in figure 4, distribution of corporate loans’ recovery rates has two peaks but it is known that the five kinds of beta estimation results (Figure 1) don’t fit the bimodal distribution. When it comes to distribution of corporate bonds’ recovery rates, it is obvious that the multiple-peaks in Figure 5 are beyond the ability of the Beta estimation which can only simulate one peak. So it can’t be a perfect tool to depict bimodal and multimodal distributions of the defaulted corporate recovery rates. The Gaussian kernel density estimation is introduced to solve this problem.

### 3. Kernel Density Estimation of the Recovery Rate

**Figure 6:**
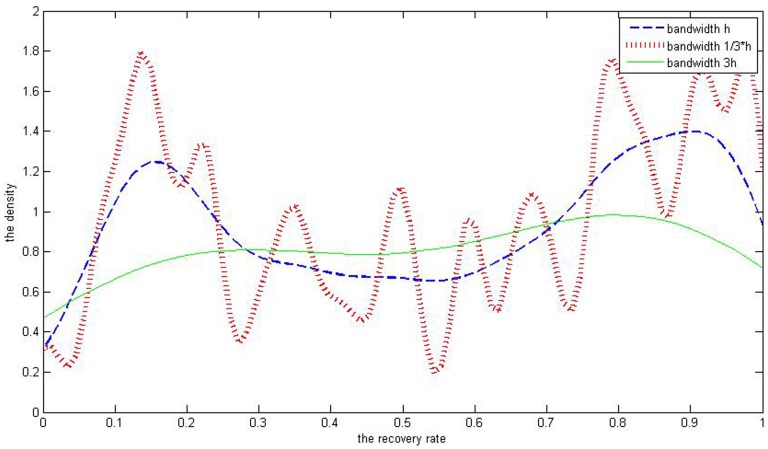
The blue is the kernel density estimation of defaulted corporate loans’ recovery rates with bandwidth of 0.0624. The green curve’s bandwidth is 0.1873.The red one is 0.0208.

The finger rule has been used in the analysis of recovery rates of bank loans, see Servigny [1] for example. It is stated as:
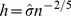
(12)where 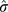 is the sample standard deviation and *n* is the observed number. In this paper, the finger rule is adopted to estimate the bandwidth we need and then we put the value of *h* into Equation (6). As to defaulted corporate loans’ recovery rates, the number of observations is 58 and the standard deviation is 0.3168. So the bandwidth *h* of this sample is 0.0624, derived through the finger rule. The bandwidth of the kernel is a free parameter which exhibits a strong influence on the resulting estimator. To illustrate its effect, the three kinds of Gaussian kernel density estimations are demonstrated in Figure 6 with bandwidths of 0.0208, 0.0624 and 0.1873 respectively.

**Figure 7:**
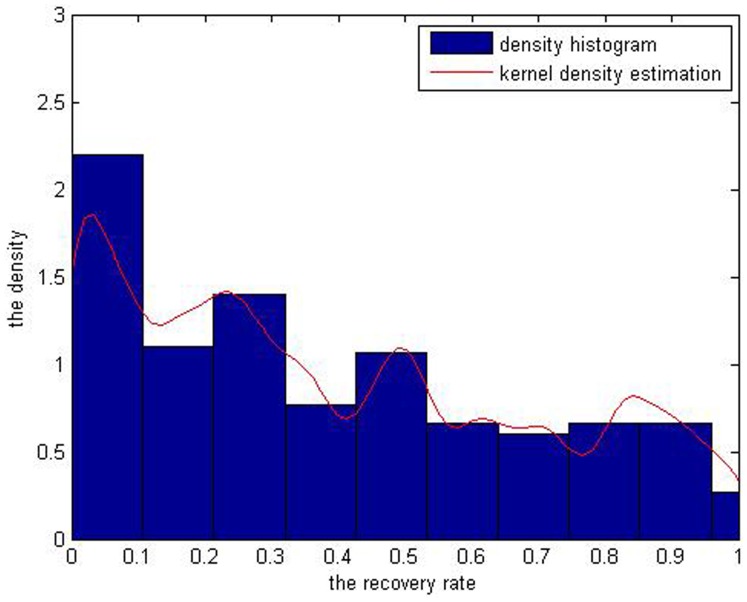
The red curve is the simulation by kernel density estimation with bandwidth of 0.0314.

Figure 6 leads to a conclusion that the larger the bandwidth is, the smoother the curve is. The green curve with the largest bandwidth is much flatter than the other two with 1/3 *h* and *h*. The curve with bandwidth of 1/*h* is much steeper and exhibits multiple peaks while the one with 3 *h* is too flat to depict the bimodal characteristics of corporate loans’ recovery rate. So the curve with bandwidth *h* best demonstrates the recovery rate’s distribution with two peaks and this proves that the bandwidth selection method we choose is appropriate. As for defaulted corporate bonds, number of observations is 282 and standard deviation is 0.2997, so the bandwidth h is 0.0314. Through Matlab7.6 software, we get its fitting curve (Figure 7). As we can see, the bonds’ recovery rates exhibit several peaks according to its density histogram. Combining the kernel density estimation curve into it, we find that the curve of the kernel density estimation has perfect fit to the distribution of the bonds’ recovery rate.

### 4. The Comparison of Gaussian Kernel Density Estimation and Beta Distribution Estimation of the Recovery Rates

**Figure 8:**
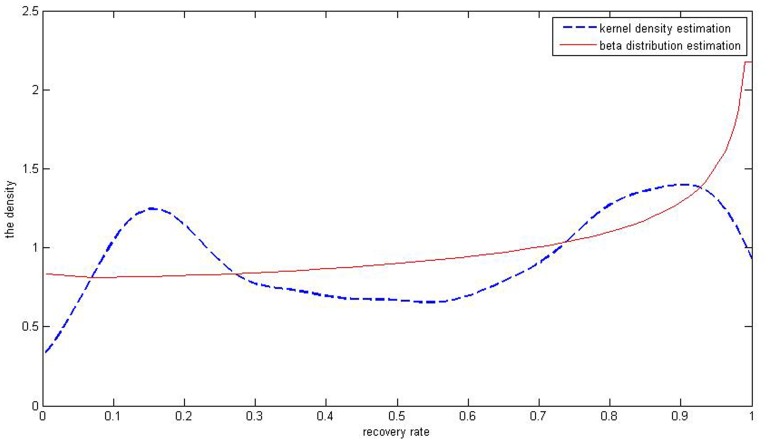
The red curve is the *J* shaped through the Beta distribution estimation of recovery rates of corporate loans. The blue one is simulated by the kernel density estimation.

**Figure 9:**
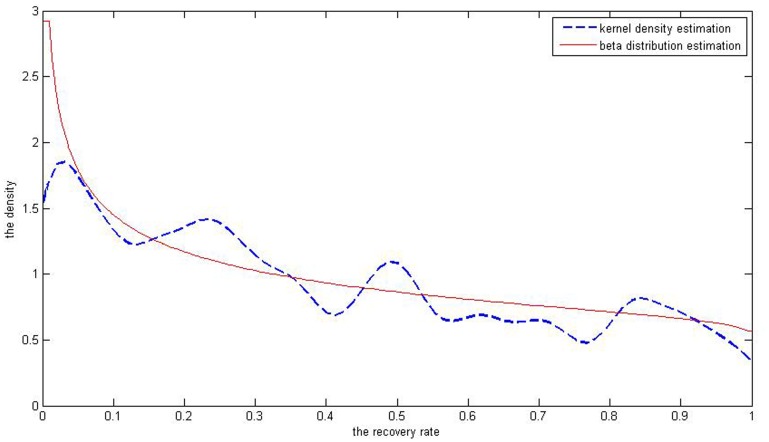
The red curve is the inverse *J* shaped through the Beta distribution estimation of recovery rates of corporate bonds. The blue one is simulated by the kernel density estimation.

Figure 8 and Figure 9 illustrate the differences between the two methods in terms of fitting of the curve to recovery rates of corporate loans and bonds.

In Figure 8, compared with Beta distribution estimation, Gaussian kernel density distribution estimation result exhibits two peaks while the Beta distribution estimation results only show a *J* shape curve, which contradicts the original data of recovery rates. And also in Figure 9, the beta distribution estimation result is an inverse *J* curve which cannot depict the multimodal distribution of recovery rates. So the kernel density estimation really better fits the distributions of the bimodal or multimodal samples with data of recovery rates of corporate loans and bonds.

### 5. Chi-square Goodness-of-fit Test Result

To assess whether the qualitative data fit the kernel density distribution, a Chi-square goodness-of-fit test is used. In the case of recovery rates of corporate loans and bonds, effective observations for the two samples are 58 and 282. At the same time, we choose 5 bins and 10 bins for each sample, because there exists one variable *h*, their degrees of freedom are 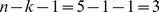 and 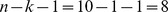 respectively. We get the results in Table 3 and Table 4 by R.

**Table 3:** Chi-square test of the estimation of recovery rates of defaulted corporate loans.

i	1	2	3	4	5
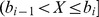	(0,0.2]	(0.2,0.4]	(0.4,0.6]	(0.6,0.8]	(0.8,1]
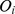	12	10	8	10	18
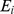	10.8417	9.8286	7.8864	10.9997	18.4438

Pearson’s Chi-squared test.

X-squared = 0.227238, df = 3, p-value = 0.973077.

**Table 4:** Chi-square test of the estimation of recovery rates of defaulted corporate bonds.

i	1	2	3	4	5	6	7	8	9	10
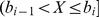	(0,0.1]	(0.1,0.2]	(0.2,0.3]	(0.3,0.4]	(0.4,0.5]	(0.5,0.6]	(0.6,0.7]	(0.7,0.8]	(0.8,0.9]	(0.9,1]
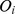	64	31	40	26	25	21	18	15	24	18
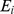	48.919	37.663	39.417	28.195	25.881	23.454	19.426	16.379	22.39	20.276

Pearson’s Chi-squared test.

X-squared = 6.13297, df = 8, p-value = 0.63234.

As we can see in Tables 3 and 4, p-values are larger than 5% significance level, so we can not reject the null hypothesis. It means that the kernel density estimator can quite fit the curve to recovery rates of corporate loans and bonds.

## Discussion

Our results find that the distribution of recovery rates display a new characteristic in the recent data and offer a new method, kernel density estimation, to estimate the distribution of the recovery rates. As we all know, the recovery rate is essential for managers and government officials to estimate the loss of their portfolios and the amount of insurance reserves. To ignore the randomness of the recovery rate by assuming it to be a constant may lower the default risk. Nowadays, financial agents have started paying more attention to management of recovery rates.

We find that corporate loans’ recovery rates follow a bimodal distribution and corporate bonds’ recovery rates have multiple-peaks. In the past years, especially before 2008, since economies were growing well, there were not too many default events, so the default probability was low and recovery rates were quite high. However, since the outbreak of the financial crisis, default events have increased, different individuals have different abilities to repay the debts. So the data we study shows a bimodal and multimodal characteristics.

We find the former two common ways to estimate the recovery rates are out of use because of the new characteristic of the recovery rates, so we introduce a new kernel density estimation to overcome it. In extant research, people always treat it as a statistical category and relatively haven’t paid too much attention. In our paper we test the two common ways on research of the distribution of recovery rate and then raise the new method, Gaussian Kernel density estimation. One is to utilize statistical methods such as histogram to estimate the distribution. The other is to parametrically estimate its density function that is set initially. Beta distribution is the most common distribution assumption in many models such as CreditMetrics by J.P.Morgan, Portfolio Manager by KMV and Losscalc by Moody’s. Combining with the data from Moody’s investor services, we find that histogram is a rough depiction and Beta distribution estimation has a main defect that it loses efficacy with the bimodal and multimodal curves. However, the Gaussian kernel density estimation, a nonparametric estimation methodology, offsets the imperfection. Finally, we prove this perspective and testify the hypothesis by the Chi-square test.

So by statistical simulation of the distribution of recovery rates with the data from Moody’s investor report, we compare the kernel density estimation with Beta distribution. We find that the kernel density estimation is better than the Beta distribution estimation while the data show bimodal or multiple-peaked characteristics, especially when the economy is in distress. And while the default events are increasing, the kernel density estimation will be beneficial for supervisors to precisely evaluate the loss of portfolio and allocate economic capital.

## References

[1] Arnaud DS, Olivier R (2004) Measuring and Managing the Credit Risk. New York: McGraw-Hill Companies.

[2] Wang GD, Zhan YR (2011) The double Beta model of recovery rate in credit risk management. Chinese Journal of Management Science 6 (12): 10–14.

[3] Hu Y, Perraudin W (2002) The dependence of recovery rates and defaults. Working paper, Birkbeck College.

[4] Rosch D, Scheule H (2005) A multifactor approach for systematic default and recovery risk. The Journal of Fixed Income 15(2): 63–75.

[5] Qu WJ, Xiong GJ (2011) Analysis and Application Research on Nonparametric Density Estimation. Journal of Shengyang Agriculture University 9: 468–472.

[6] Chen MZ, Chen H, Ma YC, Wang B, Tang Y, et al (2011) Recovery rate Models Based on General Beta Regression for Non-performing Loan. Journal of Applied Statistics and Management 30(5): 810–823.

[7] Frye J (2000) Depressing recoveries. Risk 11: 108–111.

[8] Pykhtin M (2003) Unexpected recovery risk. Risk 8: 74–78.

[9] Andersen L, Sidenius J (2004) Extension to the Gaussian Copula: Random recovery and random factor loadings. Journal of Credit Risk 1(1): 29–70.

[10] Dullmann K, Trapp M (2004) Systematic risk in recovery rates an empirical analysis of US corporate credit exposures. Working paper: 2.

[11] Liu HZ, He WZ (2010) Kernel density estimation of stock returns distribution and Monte Carlo simulation test- comparison study based on data after the introduction of price limit system. The world economy 2: 46–55.

[12] Zhen ZY, Li J (2011) Application of nonparametric kernel density estimation in the Hang Seng index yield distribution. Statistics and decision 9: 22–24.

[13] Shi ZL, Huang ZH (2009) Dynamic analysis of China provincial economy increase based on kernel density estimation. Economic survey 4: 60–63.

[14] Camblor PM, Alvarez JD, Corral N (2008) K-Sample test based on the common area of kernel density estimators. Journal of statistical planning and inference 138: 4006–4020.

[15] Sharon O, David C, Albert M (2011) Corporate Default and Recovery Rates, 1920–2010. Moody’s investors service: 18–21.

[16] Sharon O, David C, Albert M (2012) Corporate Default and Recovery Rates, 1920–2011. Moody’s investors service: 23–25.

[17] Martin LH (1999) An optimal local bandwidth selector for kernel density estimation. Journal of Statistical Planning and Inference 77: 37–50.

[18] Mugdadia AR, Ibrahim A (2004) A bandwidth selection for kernel density estimation of functions of random variables. Computational Statistics & Data Analysis 47: 49–62.

[19] Su JM, Zhang LH (2004) Application of Matlab tool kit. Beijing: Publishing House of Electronics Industry.

